# Clinical value of serum squamous cell carcinoma antigen levels in predicting chemosensitivity, lymph node metastasis, and prognosis in patients with cervical squamous cell carcinoma

**DOI:** 10.1186/s12885-020-06934-x

**Published:** 2020-05-14

**Authors:** Peng Chen, Liang Jiao, Fang Ren, Dan-Bo Wang

**Affiliations:** 1 Department of Obstetrics and Gynecology, Shengjing Hospital of China Medical University, Shenyang, Liaoning 110004 P.R. China; 2 Department of Gynecology, Cancer Hospital of China Medical University, Shenyang, Liaoning 110042 P.R. China

**Keywords:** Cervical squamous cell carcinoma, Serum squamous cell carcinoma antigen, Chemosensitivity, Lymph node metastasis, Prognosis, Neoadjuvant chemotherapy

## Abstract

**Background:**

Our aim was to investigate the value of serum squamous cell carcinoma (SCC) antigen levels in predicting chemosensitivity, lymph node metastasis, as well as prognosis in patients with cervical squamous cell carcinoma who received neoadjuvant chemotherapy (NACT).

**Methods:**

This retrospective study enrolled 103 patients with cervical squamous cell carcinoma and then compared the SCC antigen levels between patients who underwent NACT followed by radical surgery (NACT group) and those who underwent radical surgery alone (conventional group), and a correlation analysis between SCC antigen levels and chemosensitivity, lymph node metastasis, or survival time was conducted.

**Results:**

The SCC antigen levels changed after NACT and were associated with chemosensitivity. Moreover, the optimal cut-off value of the percentage decrease in SCC antigen level after the first chemotherapy (FSCC (%)) was 42.0%, which could be used for assessment of chemosensitivity. The rate of positive lymph nodes in patients with pretreatment SCC antigen levels ≥3.9 ng/mL was significantly decreased after NACT. The overall survival (OS) of NACT group was significantly longer than that of conventional group when the pretreatment SCC antigen levels were ≥ 4.55 ng/mL. The OS and progression-free survival rates of patients with SCC antigen levels < 2.7 ng/mL were longer than those ≥2.7 ng/mL after the first chemotherapy.

**Conclusions:**

The 42.0% of FSCC (%) after NACT is a reliable indicator of chemosensitivity. Pretreatment and posttreatment SCC antigen levels can be used in evaluating the lymph node metastases and prognosis of patients with cervical squamous cell carcinoma.

## Background

Cervical cancer is the second leading cause of cancer-related deaths in women worldwide [1, 2]. The squamous cell type accounts for > 85% of cervical cancers [3]. The primary treatments for cervical cancer are surgery and chemoradiotherapy. Recently, neoadjuvant chemotherapy (NACT) is an effective treatment, and its use has gained more attention. Compared with radical hysterectomy alone, NACT with cisplatin and taxol followed by radical surgery prolongs the disease-free survival of patients with stage II cervical squamous cell carcinoma with a large mass [4]. NACT compared with radiotherapy alleviates psychosexual dysfunction and improves the quality of life [5]. In addition, NACT can decrease the size of preoperative tumors and reduce the risk of lymph node metastasis [6]. However, the National Comprehensive Cancer Center clinical practice guidelines in oncology: cervical cancer (2018.V1) significantly influence the practice of clinicians in providing NACT before surgery due to the lack of references. Therefore, for patients who choose radical hysterectomy, predicting whether NACT should be applied before surgery and evaluating the efficacy of NACT are the key points for clinicians.

Squamous cell carcinoma (SCC) antigen belongs to the serine protease inhibitor (Serpin) family of proteins that have been confirmed as tumor markers for cervical squamous cell carcinoma [7–9]. At the time of diagnosis, the serum concentration of SCC antigen is correlated with the tumor stage, parametrial invasion, and lymph node metastasis [10, 11]. Moreover, serum level of SCC antigen may be used for monitoring response to treatment in patients with cervical cancer [12–15]. Notably, in 46–92% of patients who experience recurrence, the elevated level of SCC antigen after treatment was observed before the clinical manifestation of relapse, with a median lead time of 2–8 months [12, 13, 16–18]. Furthermore, NACT treatment is more likely to change SCC antigen levels, and response to NACT is related to the posttreatment SCC antigen level of cervical cancer [19]. However, reports about the clinical value of serum levels of SCC antigen in the assessment of response to NACT are limited. In addition to the inconsistent cutoff values of SCC antigen levels that are used to predict lymph node metastases or to diagnose recurrence in different studies [20–22], further assessment of whether the SCC antigen levels could be usedfor guiding NACT before radical surgery and the identification of the optimal cutoff value of SCC antigen levels are still significant for the diagnosis, prognosis and treatment of cervical squamous cell carcinoma.

To explore the clinical value of SCC antigen levels in guiding patients in selecting a treatment plan, evaluating the sensitivity of NACT, and predicting postoperative survival, this retrospective study compared the serum SCC antigen levels between patients with cervical squamous cell carcinoma who underwent NACT followed by radical surgery and those who underwent radical surgery alone, and an correlation analysis between SCC antigen levels and chemosensitivity, lymph node metastasis, and prognosis (overall survival [OS] and progression-free survival [PFS]) was conducted. Our findings will provide a theoretical basis for designing personalized treatment options for this disease.

## Methods

### Patients and study design

This retrospective study was approved by the ethical committee of Shengjing Hospital of China Medical University. In total, 103 patients with stage IB2 and IIA2 cervical squamous cell carcinoma who were admitted to Shengjing Hospital of China Medical University were enrolled in the present study. The diagnosis of cervical squamous cell carcinoma was pathologically confirmed via biopsy before surgery, and all patients did not receive any treatments before admission. After knowing the treatment plan, the patients chose their own treatment and were divided into the NACT (*n* = 64) and conventional (*n* = 39) groups. Patients in the NACT group underwent NACT + extensive hysterectomy + pelvic lymph node dissection. NACT was conducted as follows: two cycles of intravenous infusion of 75 mg/m^2^ docetaxel for 1 h on day 1 and infusion of 25 mg/m^2^ cisplatin for 1–3 h on days 1–3 (DC chemotherapy) were administered at 21-day intervals before surgery. Patients in the conventional group only underwent extensive hysterectomy + pelvic lymph node dissection. Meanwhile, patients in the two groups all received supplemental radiotherapy with a total dose of 40–50 Gy postoperatively, and Intensity Modulated Radiation Therapy (IMRT) was selected. Patients with other gynaecological tumours, squamous cell tumours, or benign and malignant diseases, such as benign skin diseases, lung diseases, and renal dysfunction, were excluded from the study.

Before treatment, 3–4 mL of blood samples was obtained from the patients after an early morning fast. After centrifugation, serum was collected for the chemiluminescence detection of SCC using an automated immunoassay analyser (Roche Modular E170, Roche Diagnostics, Mannheim, Germany). Postoperative specimens were submitted for pathological examinations to determine lymph node metastasis. The patients’ survival rate was obtained via telephone follow-up.

### Evaluation of chemotherapy response

In accordance with the Response Evaluation Criteria in Solid Tumors guideline, the maximum length (cm) of the cervical lesion was identified to evaluate sensitivity to chemotherapy. Briefly, all patients underwent the same kind of imaging technique, such as magnetic resonance imaging (MRI). The maximum length of tumour measured on MRI was measured by two experienced imaging physicians. Tumour response to NACT was defined as follows: complete response (CR): the lesion disappeared completely, and the short axis of the lymph nodes was < 10 mm; partial response (PR): the maximum length of the lesion was decreased by 30%; stable disease (SD): the condition is between PR and PD; and progressive disease (PD): the maximum length increased by at least 20%, and the absolute value of the maximum length was not < 5 mm. CR + PR was considered as chemotherapy-sensitive, and PD + SD was defined as chemotherapy-insensitive. Notably, the size of lymph nodes was not evaluated preoperatively. The positive lymph nodes were confirmed and counted via postoperative pathology.

### Statistical analysis

Statistical analysis was performed using the SPSS version 19.0 (SPSS Inc., Chicago, IL, USA). The n measurement data was tested for normal distribution using the one-sample Kolmogorov–Smirnov test. Data with normal distribution were presented as mean ± standard deviation (SD) and compared using the independent-samples *t*-test and one-way ANOVA for between the two groups and among several groups, respectively. If not normal distribution, data were expressed as median (interquartile range), and significant differences between the two groups or among several groups were analysed using the Mann–Whitney *t*-test and Kruskal–Wallis (H) test. The difference in the expression of SCC between any two groups of patients before chemotherapy, after the first chemotherapy, and after the second chemotherapy was tested using the paired *t*-test. The qualitative data were represented as n (%), and compared using chi-square (χ2) test. In the correlation analysis, Pearson correlation analysis was adopted if the two variables were continuous and conformed to a normal distribution; otherwise, Spearman correlation analysis was conducted. The predictive value and chemosensitivity of SCC antigen were analysed using the receiver operating characteristic (ROC) curve. ROC curve is a popular statistical tool for evaluating the performance of a diagnostic test, which depicts the trade-off between the sensitivity and (1-specificity) across mulitiple cut-off points [23, 24]. Survival analysis for the comparison of OS and PFS between different treatments was conducted using the Kaplan–Meier method, followed by log-rank test for difference analysis. A *P* value < 0.05 was considered statistically significant.

## Results

### Clinical characteristics of patients

The characteristics of patients with cervical squamous cell carcinoma are shown in Table 1. In accordance with the Response Evaluation Criteria in Solid Tumors guideline, the tumor response to NACT in 1, 30, 32, and 1 patient in the NACT group was defined as CR, PR, SD, and PD, respectively. Therefore, 31 patients were divided into the chemotherapy-sensitive group, and 33 patients were assigned into the chemotherapy-insensitive group. Results showed no significant differences in these clinical characteristics between the NACT and conventional groups as well as between the chemotherapy-sensitive and chemotherapy-insensitive groups.

**Table 1:** Clinical characteristics of patients with cervical squamous cell carcinoma

Clinical characteristics	NACT group _(*n* = 64)_	Conventional group (*n* = 39)	t/χ^2^	P	Chemotherapy-insensitive group (*n* = 33)	Chemotherapy-sensitive group (*n* = 31)	t/χ^2^	P
Age	47.25 ± 8.45	47.97 ± 8.23	− 0.422	0.674	47.30 ± 6.83	47.19 ± 10.00	0.051	0.960
Pathological grade			0.840	0.657			6.142	0.057
G1	16 (25.0)	13 (33.3)			7 (21.2)	9 (29.0)		
G2	41 (64.1)	22 (56.4)			25 (75.8)	16 (51.6)		
G3	7 (10.9)	4 (10.3)			1 (3.0)	6 (19.4)		
Lymph nodes			1.114	0.391			1.502	0.220
Positive	26 (40.6)	20 (51.3)			11 (33.3)	15 (48.4)		
Negative	38 (59.4)	19 (48.7)			22 (66.7)	16 (51.6)		
The number of positive lymph nodes	1.0 (1.0, 4.0)	1.0 (1.0, 3.0)	−0.012	0.991	1.0 (1.0, 3.0)	1.0 (1.0, 4.0)	−0.339	0.734
FIGO			3.232	0.072			0.119	0.730
Ib2	22 (34.4)	7 (17.9)			12 (36.4)	10 (32.3)		
IIA2	42 (65.6)	32 (82.1)			21 (63.6)	21 (67.7)		
Lesion size	5.10 ± 1.40	5.12 ± 0.74	−0.061	0.944	4.86 ± 1.47	5.36 ± 1.31	−1.419	0.161

### Comparison of SCC antigen levels between different treatments

**Fig. 1:**
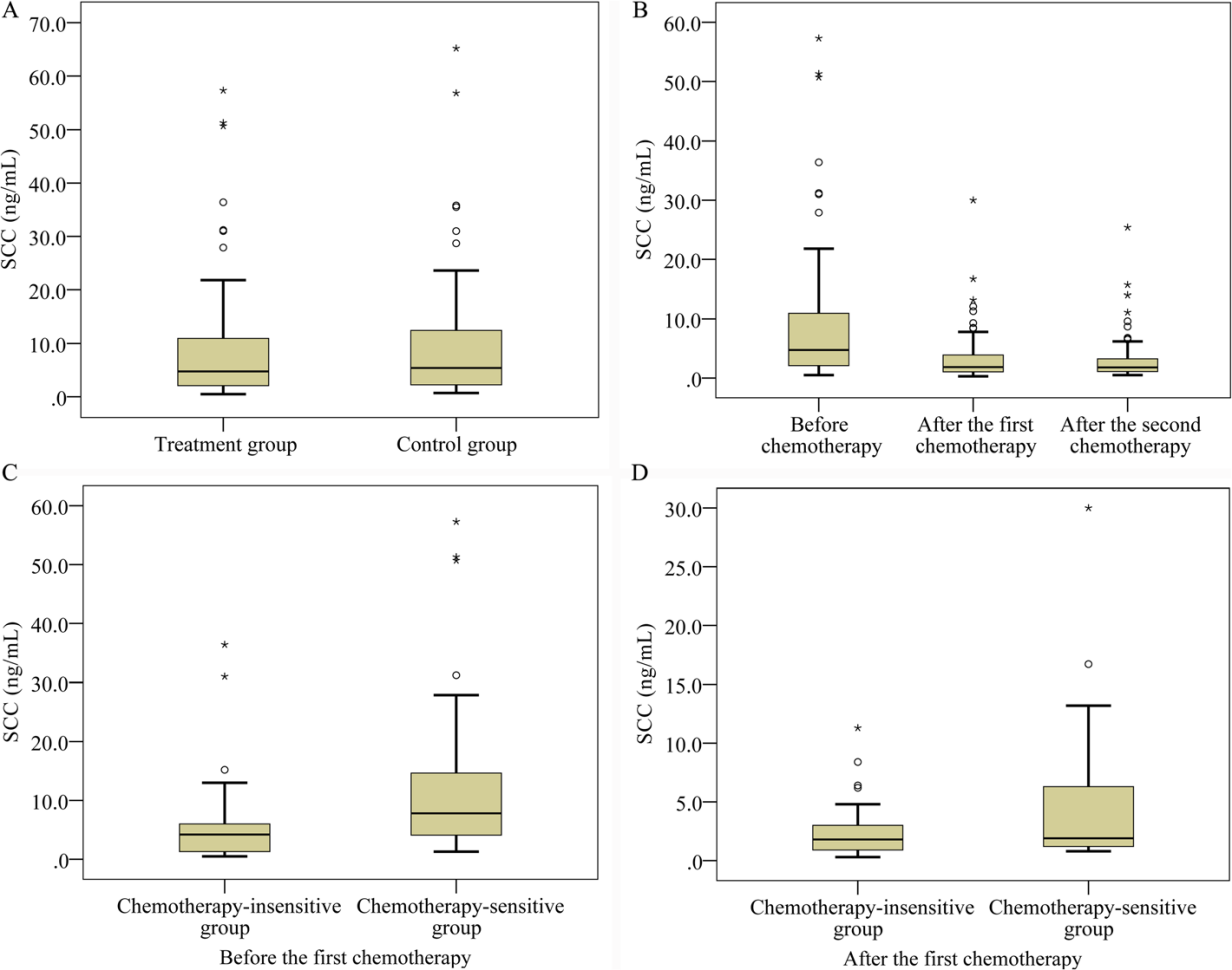
Comparison of serum squamous cell carcinoma (SCC) antigen levels between different treatments. **a** The SCC antigen levels of patients in the NACT and conventional groups; **b** The SCC antigen levels of patients in the NACT group before chemotherapy, after the first chemotherapy and before surgery; **c** and **d** The SCC antigen levels of patients in the chemotherapy-sensitive and chemotherapy-insensitive groups before and after the first chemotherapy

As shown in Fig. 1a, the SCC antigen levels expressed as median (interquartile range [IR]) were 4.75 (2.05, 10.98) ng/mL and 5.40 (2.20, 13.60) ng/mL in the NACT and conventional groups without significant difference (Z = − 0.377, *P* = 0.706). Moreover, the concentrations of SCC antigen in the NACT group were 4.75 (2.05, 10.98), 1.85 (1.03, 4.00), and 1.80 (1.10, 3.30) ng/mL before chemotherapy, after the first chemotherapy and after the second chemotherapy, respectively (Fig. 1b), and the overall difference among the three treatment time points was statistically significant (χ2 = 25.144, *P* <  0.001). Moreover, there were distinct differences in the SCC antigen levels between before chemotherapy and after the first chemotherapy (t = 5.159, *P* <  0.001) as well as before chemotherapy and after the second chemotherapy (t = 4.607, *P* <  0.001). In addition, the SCC antigen concentrations of patients in the chemotherapy-sensitive group (7.80 [4.10, 15.80] ng/mL) were visibly higher than those in chemotherapy-insensitive group (4.20 [1.20, 6.90] ng/mL) before the first chemotherapy (Z = − 2.781, *P* = 0.005, Fig. 1c). However, no remarkable differences were observed in terms of the SCC antigen levels between chemotherapy-sensitive (1.90 [1.20, 6.80] ng/mL) and chemotherapy-insensitive (1.80 [0.90, 3.25] ng/mL) groups after the first chemotherapy (Z = − 1.392, *P* = 0.164, Fig. 1d).

### Analysis the correlation between SCC antigen levels and the clinical characteristics of patients with cervical squamous cell carcinoma

As shown in Table 2, the correlation between levels of SCC antigen and clinical characteristics of patients was investigated. In the NACT group, the SCC antigen level before the first chemotherapy (ISCC), The SCC antigen level after the first chemotherapy (SSCC), the SCC antigen level after the second chemotherapy (OSCC), the absolute value of the decreased SCC antigen level after the first chemotherapy (FSCC), the absolute value of the decreased SCC antigen level after total chemotherapy (TSCC), percentage decrease in SCC antigen level after the first chemotherapy (FSCC (%)) and percentage decrease in SCC antigen level after total chemotherapy (TSCC (%)) were assessed. Results showed the lack of correlation between SCC antigen level as well as age, degree of tumor differentiation, and lesion size. However, the number of positive lymph nodes was significantly correlated to SCC antigen level in the conventional group (*P* <  0.001), as well as ISCC (*P* = 0.009), SSCC (*P* < 0.001), OSCC (*P* < 0.001), and TSCC (*P* = 0.029) in the NACT group; the maximum length of the lesion before chemotherapy was significantly correlated to SCC antigen level in the conventional group (*P* < 0.001), as well as ISCC (*P* = 0.004), FSCC (*P* < 0.001), TSCC (*P* = 0.001), and FSCC (%) (*P* = 0.002) in the NACT group. Moreover, the narrowing extent of the lesion was clearly related to ISCC (*P* = 0.008), FSCC (*P* = 0.001), TSCC (*P* < 0.001), FSCC (%) (*P* = 0.002), and TSCC (%) (*P* < 0.001), and the survival time was remarkably correlated to SSCC (*P* = 0.037) and OSCC (*P* = 0.037).

**Table 2:** Correlation analysis of SCC antigen levels with clinical characteristics of patients with cervical squamous cell carcinoma

Clinical characteristics		Conventional group	NACT group						
		SCC antigen	ISCC	SSCC	OSCC	FSCC	TSCC	FSCC (%)	TSCC (%)
Age	Spearman CC	0.204	0.064	0.095	0.134	0.073	0.037	0.102	0.057
	*P* value	0.220	0.616	0.457	0.295	0.565	0.774	0.421	0.655
Tumor differentiation degree	Spearman CC	0.076	0.111	0.195	0.117	0.109	0.158	−0.016	0.126
	*P* value	0.644	0.381	0.123	0.359	0.389	0.212	0.903	0.323
Number of positive lymph nodes	Spearman CC	0.673	0.326	0.436	0.395	0.237	0.274	0.060	0.089
	*P* value	< 0.001	0.009	< 0.001	0.001	0.059	0.029	0.637	0.484
Maximum length of the lesion before chemotherapy	Spearman CC	0.643	0.352	0.217	0.212	0.429	0.402	0.383	0.283
	*P* value	< 0.001	0.004	0.085	0.096	< 0.001	0.001	0.002	0.023
Maximum length of the lesion after chemotherapy	Spearman CC	–	0.046	0.142	0.183	0.019	0.040	−0.020	−0.075
	*P* value	–	0.718	0.263	0.151	0.880	0.754	0.874	0.558
Narrowing extent of the lesion	Spearman CC	–	0.326	0.083	0.003	0.413	0.391	0.387	0.424
	*P* value	–	0.008	0.515	0.980	0.001	0.001	0.002	<0.001
Survival time	Spearman CC	−0.060	−0.154	−0.263	−0.265	−0.086	− 0.030	0.033	0.111
	*P* value	0.716	.0.227	0.037	0.037	0.502	0.816	0.800	0.387

*CC* Correlation Coefficient, *SCC antigen* Squamous cell carcinoma antigen, *ISCC* The SCC antigen level before the first chemotherapy, *SSCC* The SCC antigen level after the first chemotherapy, *OSCC* The SCC antigen level after the second chemotherapy, *FSCC* The absolute value of the decreased SCC antigen level after the first chemotherapy, *TSCC* The absolute value of the decreased SCC antigen level after total chemotherapy, *FSCC (%)* Percentage decrease in SCC antigen level after the first chemotherapy, *TSCC (%)* Percentage decrease in SCC antigen level after total chemotherapy

### Correlation analysis of SCC antigen levels with chemosensitivity in NACT group

**Fig. 2:**
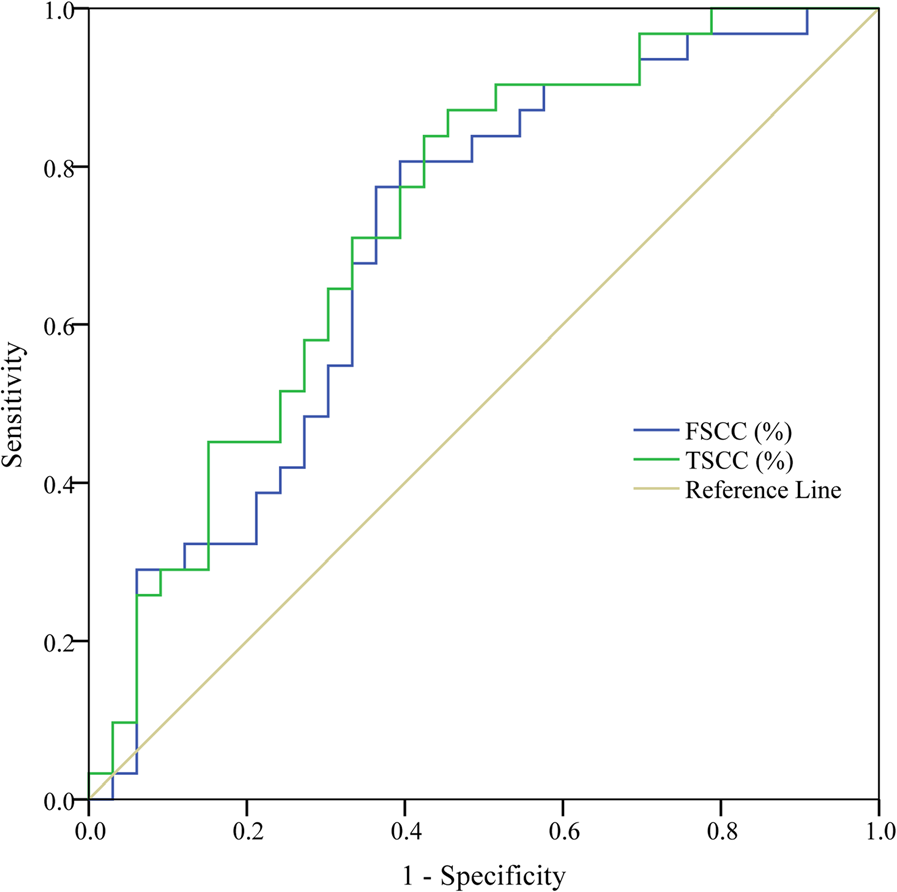
Receiver operator characteristic (ROC) curve analysis showed the value of the percentage decrease in of SCC antigen levels in predicting the chemosensitivity of patients in the NACT group. FSCC (%): Percentage decrease in SCC antigen level after the first chemotherapy; TSCC (%): Percentage decrease in of SCC antigen level after total chemotherapy

As shown in Fig. 2, ROC curve analysis revealed that the area under the curve (AUC) values of FSCC (%) and TSCC (%) were 0.702 (0.572, 0.831) and 0.732 (0.609, 0.855), respectively, and the optimal cut-off values were 42.0 and 37.0%, respectively. The the sensitivity, specificity, PPV, and NPV of FSCC (%) in assessing chemosensitivity were 80.6, 60.6, 65.8, and 76.9%, respectively, and those of TSSC (%) were 87.1, 54.5, 64.3, and 81.8%, respectively. According to the optimal cut-off values of FSCC (%), the patients were divided into ≥42% and < 42% of FSCC (%) groups and we further compared the chemosensitivity between two groups in both the chemotherapy-sensitive and chemotherapy-insensitive patients. Results showed that there was significantly difference in chemosensitivity between ≥42% and < 42% of FSCC (%) groups in both chemotherapy-sensitive (25 (80.6%) vs. 6 (19.4%)) and chemotherapy-insensitive (13 (39.4%) vs. 20 (60.6%)) patients (χ^2^ = 11.276, *P* = 0.001), and this result indicates that 42.0% of FSCC (%) could be used for the assessment of a patient’s chemosensitivity.

### Correlation analysis of the levels of SCC antigen with the rate of positive lymph nodes

The correlation between SCC antigen levels and the rate of positive lymph nodes was also investigated. Results showed that the AUC of the SCC of the conventional group was 0.893 (0.780, 1.000), and a statistical significance was observed. Meanwhile, the cut-off value was 6.75 ng/mL (Table 3). In the NACT group, the AUC values of ISCC, SSCC, FSCC, and TSCC in predicting the rate of positive lymph nodes were 0.677 (0.543, 0.810), 0.739 (0.610, 0.869), 0.634 (0.495, 0.773) and 0.655 (0.519, 0.791), respectively, and their optimal cut-off values were 3.90, 2.10, 1.45, and 1.55 ng/mL, respectively (Table 3). Notably, the AUC values of ISCC, SSCC, and TSCC were statistical significant.

**Table 3:** The value of SCC antigen levels on assessment of rate of positive lymph nodes and survival time of patients with cervical squamous cell carcinoma

Group	Factors	Cut-off	Sen (%)	Spe (%)	PPV (%)	NPV (%)	AUC (95%CI)	P of AUC
Positive rate of lymph nodes								
Conventional group (*n* = 39)	SCC antigen	≥ 6.75	80.0 (16/20)	100.0 (19/19)	100.0 (16/16)	82.6 (19/23)	0.893 (0.780, 1.000)	< 0.001
NACT group (n = 64)	ISCC	≥ 3.90	80.8 (21/26)	47.4 (18/38)	51.2 (21/41)	78.3 (18/23)	0.677 (0.543, 0.810)	0.017
	SSCC	≥ 2.10	73.1 (19/26)	71.1 (27/38)	63.3 (19/30)	79.4 (27/34)	0.739 (0.610, 0.869)	0.001
	FSCC	≥ 1.45	80.8 (21/26)	50.0 (19/38)	52.5 (21/40)	79.2 (19/24)	0.634 (0.495, 0.773)	0.071
	TSCC	≥ 1.55	76.9 (20/26)	57.9 (22/38)	55.6 (20/36)	78.6 (22/28)	0.655 (0.519, 0.791)	0.036
Survival times								
Conventional group (n = 39)	SCC	≤ 3.45	80.0 (4/5)	67.6 (23/34)	26.7 (4/15)	95.8 (23/24)	0.656 (0.359, 0.953)	0.266
NACT group (*n* = 63)	ISCC	≥ 4.55	100.0 (11/11)	57.7 (30/52)	33.3 (11/33)	100.0 (30/30)	0.798 (0.679, 0.917)	0.002
	SSCC	≥ 2.70	81.8 (9/11)	71.2 (37/52)	37.5 (9/24)	94.9 (37/39)	0.803 (0.667, 0.940)	0.002
	OSCC	≥ 2.75	81.8 (9/11)	80.8 (42/52)	47.4 (9/19)	95.5 (42/44)	0.839 (0.730, 0.948)	< 0.001
	FSCC	≥ 2.80	81.8 (9/11)	61.5 (32/52)	31.0 (9/29)	94.1 (32/34)	0.738 (0.585, 0.891)	0.014
	TSCC	≥ 1.35	90.9 (10/11)	46.2 (24/52)	26.3 (10/38)	96.0 (24/25)	0.677 (0.507, 0.846)	0.067

One patient in the NACT group had a lack of survival time. *ISCC* The SCC antigen level before the first chemotherapy, *SSCC* The SCC antigen level after the first chemotherapy, *OSCC* The SCC antigen level after the second chemotherapy, *FSCC* The absolute value of the decreased SCC antigen level after the first chemotherapy, *TSCC* The absolute value of the decreased SCC antigen level after total chemotherapy

The rate of positive lymph nodes of the conventional group, the NACT group before chemotherapy and the NACT group after the first chemotherapy was further compared according to whether the SCC antigen levels were greater or less than 3.9 ng/mL. Results showed that the rate of positive lymph nodes was not significantly different among the three groups regardless if the SCC antigen level is < 3.9 (χ^2^ = 1.919, *P* = 0.383) or ≥ 3.9 (χ^2^ = 5.758, *P* = 0.056), as well as between any two groups regardless if the SCC antigen level is < 3.9 or ≥ 3.9 (*P* > 0.05) (data not shown). Moreover, in the NACT group, 25 patients had SCC antigen levels ≥3.9 before chemotherapy and < 3.9 after the first chemotherapy. Among them, 9 patients had positive lymph nodes, and the rate of positive lymph nodes was 36.0% (9/25). In the conventional group, 18 patients with a SCC antigen level ≥ 3.9 ng/mL underwent surgery, and the rate of positive lymph nodes was 78.3% (18/23). A significant difference existed in the rate of positive lymph nodes between the above two groups (χ^2^ = 8.694, *P* = 0.003).

### Correlation analysis of levels of SCC antigen with survival time of patients

For the assessment of the survival times of patients, the AUC value of the SCC of the conventional group was 0.656 (0.359, 0.953). However, the result was not statistically significant, and the cut-off value was 3.45 ng/mL (Table 3). In the NACT group, the AUC values of ISCC, SSCC, OSCC, FSCC, and TSCC were 0.798 (0.679, 0.917), 0.803 (0.667, 0.940), 0.839 (0.730, 0.948), 0.738 (0.585, 0.891), and 0.677 (0.507, 0.846), respectively, and their cut-off values were 4.55, 2.70, 2.75, 2.80, and 1.35 ng/mL, respectively (Table 3). Only the TSCC did not have a statistical significance.

In terms of the OS and PFS, there were no significant differences between conventional and NACT groups (χ^2^ = 0.095, *P* = 0.758; χ^2^ = 0.054, *P* = 0.817, respectively), as well as between chemotherapy-sensitive and chemotherapy-insensitive groups (χ^2^ = 0.098, *P* = 0.754; χ^2^ = 0.0002, *P* = 0.988, respectively) (Tables 4 and 5). Moreover, we compared the OS and PFS of patients in the NACT group between SCC antigen levels < 4.55 and ≥ 4.55 ng/mL before chemotherapy, and a significant difference was observed between them (χ^2^ = 9.880, *P* = 0.002; χ^2^ = 12.148, *P* < 0.001, respectively) (Tables 4 and 5). When the SCC antigen levels were > 4.55 ng/mL before chemotherapy, the OS of the NACT group was remarkably longer than that of the conventional group (χ^2^ = 4.176, *P* = 0.041) (Table 4). However, PFS did not exhibit significant difference between the two groups (χ^2^ = 4.176, *P* = 0.071) (Table 5). Furthermore, after the first chemotherapy, the OS and PFS of patients with SCC antigen levels < 2.7 ng/mL in the NACT group was longer than those ≥2.7 ng/mL (χ^2^ = 10.869, *P* = 0.001; and χ^2^ = 13.954, *P* < 0.001) (Tables 4 and 5).

**Table 4:** Correlation analysis of SCC antigen levels with overall survival of patients with cervical squamous cell carcinoma

Variable	Mean survival times (months)	χ^2^	P
SCC antigen levels before chemotherapy		0.095	0.758
NACT group (n = 63)	40.16 (37.24, 43.07)		
Conventional group (*n* = 39)	33.43 (31.31, 35.56)		
NACT group		0.098	0.754
Chemotherapy-insensitive group (*n* = 32)	41.19 (37.29, 45.08)		
Chemotherapy-sensitive group (n = 31)	39.25 (35.01, 43.50)		
SCC antigen levels in NACT group before chemotherapy		9.880	0.002
< 4.55 (*n* = 29)	NA ^a^		
≥ 4.55 (*n* = 34)	NA ^a^		
SCC antigen levels ≥4.55 before chemotherapy		4.176	0.041
NACT group (n = 34)	36.40 (31.59, 41.22)		
Conventional group (*n* = 21)	32.95 (30.95, 34.96)		
SCC antigen levels in NACT group after the first chemotherapy		10.869	0.001
< 2.70 (n = 39)	43.75 (42.07, 45.43)		
≥ 2.70 (*n* = 24)	34.40 (28.99, 40.81)		

^a^, in NACT group before chemotherapy, when SCC antigen levels < 4.55 ng/mL, 29 patients were survival and it is impossible to calculate the survival times of patients

**Table 5:** Correlation analysis of SCC antigen levels with progression-free survival of patients with cervical squamous cell carcinoma

Variable	Mean survival times (months)	χ^2^	P
SCC antigen levels before chemotherapy		0.054	0.817
NACT group (n = 63)	38.02 (34.43, 41.61)		
Conventional group (*n* = 39)	31.99 (29.25, 34.74)		
NACT group		0.0002	0.988
Chemotherapy-insensitive group (n = 32)	36.34 (31.50, 41.19)		
Chemotherapy-sensitive group (n = 31)	38.09 (33.07, 43.12)		
SCC antigen levels in NACT group before chemotherapy		12.148	< 0.001
< 4.55 (n = 29)	NA ^a^		
≥ 4.55 (n = 34)	NA ^a^		
SCC antigen levels ≥4.55 before chemotherapy		3.256	0.071
NACT group (n = 34)	30.21 (24.74, 35.67)		
Conventional group (*n* = 22)	31.27 (28.21, 34.34)		
SCC antigen levels in NACT group after the first chemotherapy		13.954	< 0.001
< 2.70 (n = 39)	43.34 (41.09, 45.58)		
≥ 2.70 (n = 24)	24.74 (18.80, 30.68)		

^a^, in NACT group before chemotherapy, when SCC antigen levels < 4.55 ng/mL, 29 patients were survival and it is impossible to calculate the survival times of patients

## Discussion

SCC is one of the most useful tumor markers for the early diagnosis of recurrence and response to specific treatment [25]. To our best knowledge, the application of NACT for cervical cancer treatment is controversial because of the lack of references to NACT before surgery. Evaluating the efficacy of NACT and predicting whether NACT can be performed before radical hysterectomy will help improve the clinical outcomes of patients with cervical squamous cell carcinoma. In this retrospective study, we found that the SCC antigen levels changed after NACT and were associated with sensitivity to chemotherapy. Moreover, the optimal cut-off value of FSCC (%) was 42.0% for assessment of chemosensitivity. The rate of positive lymph nodes in patients with SCC antigen levels ≥3.9 ng/mL before treatment was significantly decreased after NACT. Furthermore, the OS of the NACT group was markedly longer than that of the conventional group when the SCC antigen levels were ≥ 4.55 ng/mL before chemotherapy, and the OS and PFS of patients with SCC antigen levels < 2.7 ng/mL in the NACT group were all overtly higher than those ≥2.7 ng/mL after the first chemotherapy.

A prospective cohort study has also revealed that serum level of SCC antigen is a reliable and sensitive factor in the assessment of response to chemotherapy in cervical cancer patients [26]. Hong et al. reported that persistently elevated levels of SCC antigen resulted in a greater possibility of treatment failure after 2–3 months of radiotherapy [27]. Hashimoto et al. have revealed that declining SCC antigen level is related to a good chemotherapy response in patients with metastatic cervical cancer [28]. In addition, after NACT, monitoring of SCC antigen levels could reflect the response to chemotherapy [19]. In this study, the SCC antigen levels of chemotherapy-sensitive group were significantly higher than chemotherapy-insensitive group before the first chemotherapy, suggesting that the basal concentration of SCC antigen were associated with the sensitivity to chemotherapy in patients with cervical squamous cell carcinoma. Moreover, we also found that the concentration of SCC antigen were significant different between before chemotherapy and after the first chemotherapy, and this result indicated that the SCC antigen levels changed after NACT. Notably, sensitivity to chemotherapy is correlated to decreased SCC antigen expression levels in cervical cancer patients who underwent DC chemotherapy and subsequent radical surgery [29]. Nevertheless, no study has evaluated the value of the percentage decrease in SCC antigen levels after NACT in monitoring response to the chemotherapy. For patients who are insensitive to chemotherapy, the best time for other treatments may be delayed if the efficacy of chemotherapy is evaluated after 2–3 times of NACT before surgery. In this study, although the SCC antigen levels were changed after the first chemotherapy, significant difference in SCC antigen levels was not obtained between after the first chemotherapy and after the second chemotherapy. These data indicated that patients who are sensitive to NACT can be identified after the first chemotherapy. Furthermore, we first assessed the value of FSCC (%) in the assessment of chemosensitivity, and results showed that the optimal cut-off value of FSCC (%) was 42.0% and that there was a significantly difference in chemosensitivity between the ≥42% and < 42% of FSCC (%) groups in both chemotherapy-sensitive and chemotherapy-insensitive patients. These findings indicated that 42.0% of FSCC (%) after NACT could be used as a reliable indicator in assessing chemosensitivity in patients with cervical squamous cell carcinoma.

Lymph node metastasis is a key clinical parameter in determining the treatment and prognosis of cervical cancer, which is a key factor that affects the 5-year survival rate [30, 31]. In early-stage cervical carcinoma, the 5-year of rate of lymph node-positive patients (around 50%) was significantly lower than that of lymph node-negative patients (approximately 90%) [32]. In addition, a meta-analysis and literature review summarizes the value of SCC antigen level in the determination of lymph nodal metastasis in cervical cancer [33]. Our data indicated that the number of positive lymph nodes was significantly correlated to SCC antigen level in the conventional group (ISCC, SSCC, OSCC, and TSCC) (*P* = 0.029). Furthermore, the rate of positive lymph nodes in patients with pretreatment SCC antigen levels ≥3.9 ng/mL was significantly reduced after NACT. Therefore, we speculated that NACT should be performed in patients with pretreatment SCC levels ≥3.9 ng/mL, and even if the SCC antigen levels decrease to 3.9 ng/mL after chemotherapy, it could be used as a critical indicator in predicting the sensitivity of lymph node metastases to NACT.

Furthermore, in patients with early-stage squamous cervical cancer, the elevated levels of pretreatment SCC antigen was distinct correlated with poor prognosis of patients [34]. Accumulating evidence has validated that the variation in SCC antigen levels during the treatment could influence the prognosis of patients who experienced recurrence [35, 36] Li et al. have also shown that elevated pretreatment SCC antigen levels (> 3.5 ng/mL) were correlated to a higher risk of lymph node metastases and a poor response to NACT in cervical cancer patients who received NACT and underwent radical hysterectomy [37]. In this study, OS and PFS had no obvious differences between the conventional and NACT groups, as well as between the chemotherapy-insensitive and chemotherapy-sensitive groups, suggesting that NACT might have no significant effect on improving the survival of patients with stage I/II cervical cancer. Despite these, the OS of the NACT group was remarkably longer than that of the conventional group when the SCC antigen levels were ≥ 4.55 ng/mL before chemotherapy. Therefore, we speculate that a better prognosis may be achieved when NACT is chosen followed by radical surgery compared with radical surgery in patients with pretreatment SCC antigen levels ≥4.55 ng/mL. Moreover, our results showed that the OS and PFS of patients with SCC antigen levels < 2.7 ng/mL in the NACT group were significantly longer than that of patients with SCC antigen levels ≥2.7 ng/mL after the first chemotherapy. We therefore speculate that patients with < 2.7 ng/mL of SCC antigen levels after the first cycle of NACT may also have a better prognosis than those with ≥2.7 ng/mL. This finding could provide an indicator to determine whether the patient is sensitive to chemotherapy. If insensitive, MRI can be carried out to judge whether the patient is suitable for NACT, and the inappropriate patient should change the treatment methods, which will reduce the pain and burden of the patients. Considering that the study was a retrospective investigation, the results required prospective studies for validation.

## Conclusions

In conclusion, SCC antigen levels are correlated to chemosensitivity, lymph node metastasis, and prognosis in patients with cervical squamous cell carcinoma. Monitoring of SCC antigen levels will help clinicians in designing personalized treatment options for patients with cervical squamous cell carcinoma.

## Data Availability

The datasets used and analyzed during the current study are available from the corresponding author on reasonable request.

## Abbreviations

NACT: Neoadjuvant chemotherapy
OS: Overall survival
PFS: Progression-free survival
CR: Complete response
PR: Partial response
SD: Stable disease
PD: Progressive disease
SCC: Squamous cell carcinoma
ISCC: SCC antigen levels before the first chemotherapy
SSCC: SCC antigen levels after the first chemotherapy
OSCC: SCC antigen levels after the second chemotherapy
FSCC: The absolute value of the decreased SCC antigen level after the first chemotherapy
FSCC (%): Percentage decrease in SCC antigen level after the first chemotherapy
TSCC: The absolute value of the decreased SCC antigen level after total chemotherapy
TSCC (%): Percentage decrease in SCC antigen level after total chemotherapy
